# Burden of chronic diseases in migrant workers (MW) in Singapore and impact on COVID-19: A large single-centre cohort study during the peak MW 2020 outbreak^☆^

**DOI:** 10.1016/j.jmh.2025.100365

**Published:** 2025-09-22

**Authors:** Sapna P. Sadarangani, Joshua K. Tan, Pei Hua Lee, Hanley Ho, Angela Chow, Rinkoo Dalan, Lai Gwen Chan, Thirugnanam Umapathi, Shawn Vasoo

**Affiliations:** a National Centre for Infectious Diseases, Singapore; b Department of Infectious Diseases, Tan Tock Seng Hospital, Singapore; c Lee Kong Chian School of Medicine, Nanyang Technological University, Singapore; d Department of Epidemiology and Preventive Medicine, Office of Clinical Epidemiology, Analytics and Knowledge, Tan Tock Seng Hospital, Singapore; e Department of Endocrinology, Tan Tock Seng Hospital, Singapore; f Department of Psychiatry, Tan Tock Seng Hospital, Singapore; g Department of Neurology, National Neuroscience Institute, Singapore; h Saw Swee Hock School of Public Health, National University of Singapore, Singapore

**Keywords:** Chronic diseases, Migrant workers, Health disparities, COVID-19 outbreak, Healthcare access, Public health policies

## Abstract

**Background:**

The COVID-19 pandemic unmasked systemic inequities amongst migrants globally. In Singapore, dormitory residing migrant workers (MWs) were disproportionately impacted accounting for over 54,500 COVID-19 cases (93% of all cases) in 2020. Their limited health insurance did not cover outpatient acute or chronic illness care. There is a lack of systematic data on the chronic disease prevalence amongst MWs.

**Methods:**

In this cohort study, we analysed the electronic medical data of all MWs with PCR-confirmed symptomatic COVID-19 admitted to the National Centre for Infectious Diseases (NCID), Singapore from 1st to 30th April 2020, the peak of the MW outbreak. We aimed to ascertain the prevalence of chronic diseases and their impact on COVID-19 disease severity.

**Results:**

2,498 male MWs were included. Majority (>90%) were of South Asian origin. Median age was 34 years (IQR 29-40). 247 (9.9%) had at least one chronic disease; 99 (4.0%) had diabetes mellitus (22 (22.2%) newly diagnosed), 69 (2.8%) had dyslipidaemia (26 (37.7%) newly diagnosed), and 135 (5.4%) had hypertension (21 (15.5%) newly diagnosed). While 278 (11.1%) had pneumonia, only 16 (0.6%) required oxygen or intensive/high-dependency care, with no mortality. Charlson’s score ≥ 1 (compared to 0) was associated with 80% higher odds of moderate-severe COVID-19 outcome, aOR (adjusted odds ratio) 1.83 (1.16-2.90), *P*=0.01, adjusted for age and nationality in multivariable analyses.

**Conclusions:**

Although MWs had favourable COVID-19 medical outcomes during their acute illness, our study demonstrates a considerable burden of undiagnosed chronic diseases predisposing to cardiovascular disease and highlights health disparities that should be addressed by health policies.

## Highlights

• A high proportion of chronic diseases amongst migrant workers were newly diagnosed.
• Charlson’s score ≥ 1 was associated with higher odds of moderate-severe COVID-19.
• The study highlights health disparities that should be addressed by health policies.

## 1 Introduction

The COVID-19 pandemic unmasked systemic, structural societal inequities amongst migrants and ethnic minorities worldwide. (Hanvoravongchai et al., 2020; Tai et al., 2021; Alali et al., 2021; Greenaway et al., 2021; Hayward et al., 2021) Migrant populations are often disproportionately affected with higher COVID-19-associated morbidity and mortality, with long-term economic and social impacts that further deepen disparities. These health inequities have been attributed to less than favourable social determinants of health, such as poor living conditions, limited access to health care, and increased burden of chronic diseases. (Tai et al., 2021; Greenaway et al., 2021; Paremoer et al., 2021)

Singapore is a high-income city-state with a multi-ethnic resident population of 5.7 million. During the initial stages of the COVID-19 pandemic in 2020, the country was successful at mitigating the community impact through a containment and eradication strategy. However, 54,506 confirmed COVID-19 cases (93.0 % of total COVID-19 cases) in 2020 had been among migrant workers (MWs) residing in dormitories. (Ministry of Health S. COVID-19 Situation Report, 2020) Singapore has more than 1.2 million foreign workers from neighbouring Asian countries. Of these, >310,000 are low-wage MWs in the construction, marine, and process (CMP) sectors, comprising 5.4 % of the total population. Ministry of Manpower Foreign workforce numbers (2022) approximately 200,000 MWs live in 43 purpose-built dormitories (PBD), whilst 95,000 reside in numerous factory-converted dormitories (FCD). (Ministry of Manpower S. Ministerial Statement by Mrs Josephine Teo, Minister for Manpower, 2020) In PBDs, 12 to 20 men share a room with a minimum of 4.5 m^2^ of living space per person. PBDs may have communal dining, kitchens, toilets, and other shared facilities such as grocery stores and recreation areas. (Ministry of Manpower S. Various type of housing [Internet], 2022) FCDs are industrial or warehouse developments converted into dormitories. (Ministry of Manpower S. Various type of housing [Internet], 2022)

MWs face crowded work and living conditions affecting their ability to effectively social distance. The first few COVID-19 cases amongst MWs occurred in early February 2020 and soon grew to multiple dormitory-based clusters nationwide. (Ngiam et al., 2021; Koh, 2020; IN FOCUS: Channel News Asia, 2022) The outbreak in MW dormitories from 31st March 2020 onwards required the implementation of extensive public health measures in the dormitories and throughout the nation. Singapore implemented enhanced restrictions and lockdown measures (locally known as “circuit breaker”) from 7th April 2020 to 7th July 2020. Medically supervised community recovery facilities were set up to receive stable recovering COVID-19 patients. Medical stations established at the dormitories permitted comprehensive increased testing. (Tan et al., 2021) The COVID-19 outbreak in MW dormitories was subsequently contained by August 2020. (Ministry of Health S. COVID-19 situation report, 2020)

Since 2008, MWs in Singapore could receive acute episodic care financed through government-mandated hospitalization health insurance valued up to Singapore dollar $15,000 per annum payable by the employer. Importantly, this did not include outpatient care or preventive care at the time of the 2020 COVID-19 outbreak prior to the Primary Care Plan (PCP) being introduced for MWs in the CMP sector in 2022. (Minstry of Manpower S, 2022; Rajaraman et al., 2020; Sadarangani SP et al., 2017; Ministry of Manpower, 2022; Ministry of Manpower, 2024) Prior to the PCP, their employment health screening included screening for HIV, tuberculosis (TB), and syphilis, but not chronic diseases such as hypertension and diabetes mellitus. There was no specific health delivery or financing for chronic diseases for MWs apart from local volunteer non-governmental organizations (NGOs). (Rajaraman et al., 2020) Funding for chronic diseases prior to the PCP was largely out-of-pocket or self-funded with high relative cost to their monthly wages.

The association of chronic diseases with COVID-19 outcomes has been well-described. (Hanvoravongchai et al., 2020; Islam et al., 2021) However, there is a relative paucity of data on this association amongst MWs due to the lack of systematic data on the chronic disease prevalence in this population at the time of the COVID-19 outbreak affecting MWs in 2020. There were very few opportunities to assess the health status of MWs in Singapore prior to the pandemic due to the nature of their healthcare access as described which represents a knowledge gap. In this study, we aimed to ascertain the prevalence of chronic diseases amongst the MWs admitted for COVID-19 during the peak of the outbreak in 2020 and evaluate the impact of chronic diseases on COVID-19 outcomes using secondary data. Owing to MWs facing poor access to longitudinal chronic disease care, we hypothesized that a significant proportion would have undiagnosed chronic diseases and MWs with chronic diseases would be more predisposed to severe COVID-19, including a higher need for care in the intensive care unit (ICU).

## 2 Methods

### 2.1 Study design

We performed a cohort study of all MWs aged 21 years and above, with PCR-confirmed COVID-19 admitted to our institution during the period 1st to 30th April 2020, following them up longitudinally until 30 days post-discharge. This period coincided with the peak of the outbreak when MWs with symptomatic COVID-19 were admitted. As the outbreak response strategy involved extensive testing and active case finding in the dormitories, larger numbers of patients with asymptomatic infections were admitted from May 2020 onwards. We aimed to examine the association between chronic disease and the severity of COVID-19 disease in symptomatic patients. The presence of chronic disease is as defined by our criteria and the Charlson’s score as referenced in “2.6 Chronic diseases of interest and definitions.” Hence, the aforementioned study period was chosen when symptomatic COVID-19 patients were admitted. We collected data from MWs’ entire index admission for COVID-19 and data from re-admissions (if any) within 30 days of discharge from their index admission.

### 2.2 Study setting

The study was performed at the National Centre for Infectious Diseases (NCID), the national designated centre for emerging infectious disease outbreaks including COVID-19, and co-located 1600-bed Tan Tock Seng Hospital (TTSH). At the start of the pandemic, TTSH and NCID managed a large proportion of the suspect/confirmed COVID-19 cases in the adult population. Licensed therapeutics for COVID-19 were not yet available at the time of the MW outbreak, but anti-viral agents such as remdesivir were being investigated in institution ethics review board-approved clinical trials. At that point in the outbreak, clinicians could offer off-label use of therapeutics on a case-by-case basis thought to be of some clinical benefit based on *in-vitro* activity to SARS-CoV-2 and/or other available published clinical and *in-vivo* data e.g. lopinavir-ritonavir, interferon-beta-1B, hydroxychloroquine, anti-inflammatory agents, following a risk-benefit discussion through informed consent, in addition to supportive care. (National Centre for Infectious Diseases COVID-19 therapeutics work-group, 2022) Our site worked with local volunteer NGOs for translator support, outpatient mental health services, and chronic disease management support. (Chan and Kuan, 2020) Medically-supervised isolation facilities known as COVID-19 recovery facilities were also set up who received stable recovering COVID-19 patients who still needed to complete their quarantine period per the prevailing public health policies at that time. (COVID-19 White Paper, 2024)

### 2.3 Study participants

We included skilled and semi-skilled MWs in the construction, marine, and process sectors who are in Singapore on a work permit, perform manual work, and live in communal housing such as PBDs, FCDs, or other shared accommodation.

Skilled workers or professionals from overseas countries working in Singapore on a S pass or Employment pass holding white-collared jobs earn higher salaries and live in private accommodation. (Work Passes, 2024) They were excluded from this study as we restricted our study population to MWs who were of a lower socioeconomic status.

### 2.4 Data sources

Data for patients who met the inclusion criteria were extracted from electronic medical records and included age, all diagnoses (ICD-9, ICD-10-AM), laboratory results, radiographic reports, visit details (including inpatient admission episodes, outpatient specialist clinics, and emergency visits), medications dispensed (including duration and date(s) of dispensing), specific procedure and treatment service information. All data were extracted by a data analytics team in a de-identified manner. Additionally, medical records for MWs with severe COVID-19 (i.e., required intensive care unit (ICU), high-dependency (HD) care, or supplemental oxygen) were individually reviewed by investigators P.H.L and S.P.S.

### 2.5 COVID-19 disease course and outcomes

We describe the COVID-19 clinical course based on laboratory biomarkers, radiographic pneumonia at presentation or later during admission, medical interventions, and complications. Moderate-severe COVID-19 was defined as individuals with radiographic pneumonia, including those who needed ICU/HD care or oxygen supplementation. We describe key characteristics of the clinical presentation, course, and complications for the MWs with moderate-severe COVID-19 who needed higher level of care in ICU/HD or oxygen supplementation in ward.

### 2.6 Chronic diseases of interest and definitions

The chronic diseases of interest were: (1) diabetes mellitus, (2) hyperlipidaemia (high low-density lipoprotein (LDL) and/or high triglycerides (TG)), (3) hypertension, (4) ischemic heart disease/cerebrovascular disease, (5) asthma/chronic lung disease, and (6) mental health/psychiatric conditions. Admitted patients were offered age-appropriate chronic disease screening if they had not had this done recently. This screening was part of their overall clinical care and helped to ascertain the comorbidities that may predispose to a more complicated COVID-19 disease course and contributed to the considerations for transfer from the acute care hospital setting to COVID-19 recovery facilities. For this study, an aggregate criterion was used to identify the presence of each chronic disease, specifically (1) continued prescription (≥ 2 days) of coded medications linked to a particular chronic disease (e.g. oral anti-hyperglycemic agents and long-term acting insulin for diabetes mellitus), (2) laboratory tests that are used for diagnosis where relevant (e.g. fasting plasma glucose or HbA1c fulfilling diagnostic criteria for diabetes mellitus) (Espino, 2010); (3) relevant ICD-9/ICD-10-AM discharge diagnosis codes.

We considered a condition to be pre-existing if the associated medications were prescribed within two days of hospital admission and continued upon discharge. This two-day time frame accounts for the time required to conduct medication reconciliation by the hospital pharmacist and the electronic ordering of medications for pre-existing conditions. The medication reconciliation process identified medications and supplements the MW had been consuming or was meant to be consuming (active medications) but was not currently doing so due to lack of supply, adherence, and other factors. This includes medications that had been prescribed in Singapore, such as by general practitioners, as well as medications obtained by the MW from his home country. We classified the condition as a new diagnosis if the medications for a condition were prescribed ≥ 2 days after admission and continued upon discharge.

The diagnosis of hypertension was based on discharge diagnosis and anti-hypertensive medications prescribed on discharge, and not the prescription of anti-hypertensives alone. MWs with elevated blood pressure readings during acute COVID-19 may have received antihypertensives on discharge without a discharge diagnosis of hypertension.

The Charlson’s comorbidity score was calculated for each MW. The same aggregate criterion was applied for determining diagnosis of the comorbidities included in the Charlson’s comorbidity score that were not in the list of chronic diseases of interest, such as peptic ulcer disease. The Charlson’s comorbidity score is a well-known and readily applied summative score of various chronic diseases that predict long term mortality. Additionally, in relation to COVID-19 related outcomes, Kuswardhani et al. described in their systematic review and meta-analysis that a higher Charlson’s comorbidity score compared to score of 0 was associated with COVID-19 related mortality and morbidity. (Kuswardhani et al., 2020)

### 2.7 Statistical analyses

Continuous variables are described by median, IQR, and range, and categorical variables by counts or proportions. Continuous variables were evaluated for normality using the Shapiro-Wilk test. Though there were few continuous variables that were normally distributed, the median and IQR have been used as the summary statistic for all continuous variables for consistency. Statistical tests of significance for comparisons between categorical variables were by chi-squared test including chi-squared test for trend where relevant and continuous variables via *t*-test (normally distributed variables) or by Mann-Whitney U test (for skewed outcome variables).

We compared demographic and clinical parameters among MWs with at least one chronic disease with MWs without any known chronic disease in bivariate analyses. Statistically significant factors known *a priori* to contribute to severe COVID-19 disease from the bivariate analyses were included in the multivariable analysis. Charlson’s score was used to represent the presence of chronic disease, the exposure of interest. (Kuswardhani et al., 2020) A multivariable logistic regression model was designed to assess associations of Charlson’s score (score of ≥ 1 versus 0), age, and nationality (Bangladeshi, Indian, and others) with moderate-severe COVID-19 outcome. Fifty-four patients who did not have a chest radiograph during the index admission and were discharged well were classified as having mild COVID-19. Multi-collinearity was assessed by variance-inflation factor (VIF) of variables included in the model. The mean VIF was 3.62 hence there was no significant multi-collinearity. Data analyses were performed using Stata version 16.0 (StataCorp, College Station, TX).

### 2.8 Ethics

Ethics approval was obtained from the National Healthcare Group (NHG) Domain Specific Review Board before initiating this study (NHG DSRB reference number: 2020/00,863) which approved a waiver for informed consent.

## 3 Results

### 3.1 Study population and initial outcomes of COVID-19

A total of 2748 patient admission encounters during the study period were screened and 2498 MWs were included. All MWs were male with a median age of 34 years (IQR 29–40), mean age 34.9 years (SD 7.8) and range 21–62 years (Table 1). The majority had originated from Bangladesh and India with smaller proportions from China, Thailand, Malaysia, Myanmar, and Vietnam. Most (97.6 %) MWs lived in dormitory-type housing. The median length of stay for the index COVID-19 admission was 5 days (IQR 4–6) and range 2–66 days. While 278 (11.1 %) had pneumonia on presentation, only 16 (0.6 %) required oxygen or ICU/HD care, with no mortality. Fifty-three (2.1 %) required re-admission within 30 days of discharge from the index admission. Table 2 shows the detailed synopsis of the clinical course for the 16 patients who needed a higher level of care; there were 10 episodes of ICU/HD care (62.5 %) with median ICU LOS of 3.5 [IQR 3.0–8.0] days. Thirteen (81.3 %) patients received oxygen supplementation either in the ward or in ICU/HD and six patients (37.5 %) received anti-viral medications for COVID-19. The most common reason for ICU/HD care was respiratory failure for 6 patients, cardiac monitoring for 3 patients and 1 patient required admission for both indications. Only 3 patients (18.8 %) had known pre-existing comorbidities (Table 2).

**Table 1:** Demographic and clinical descriptive variables for MW’s index COVID-19 admission and prevalence of chronic diseases, *n* = 2498.

**Demographic/Descriptive Characteristics**	**Sub-categories where relevant**	**N or median**	**% or IQR**
Age (years)	–	34	29–40
Nationality	Bangladeshi	1659	66.4
	Indian	634	25.4
	Chinese	111	4.4
	Thai	18	0.7
	Others	76	3.0
Housing (dormitory or private housing)	Dormitory	2439	97.6
	Non-dormitory private	59	2.4
Charlson’s Co-morbidity Index score	–	0	0–0 Range: 0–4
Length of stay for index hospital admission for COVID-19	–	5	4–6
Required higher level of care either ICU/HD or supplementary oxygen supplementation in general ward (Yes)	Overall count	16	0.6
	ICU or HD care (unique patients)	9	0.4
	Supplementary oxygen in the general ward alone	7	0.4
**COVID-19 related radiology**	**Tests done**	**N**	**% (denominator is tests performed)**
Abnormal CXR within the first 2 days of admission	2444	278	11.3
Abnormal CT thorax during admission	17	7	41.2
Normal CXR within the first 2 days of admission with subsequent abnormal CXR	2166	29	1.3
**COVID-19 related biomarkers**	**Tests done**	**Median**	**IQR**
Absolute lymphocyte count (ALC) on admissionx10^9^/L Laboratory reference range for normal: 1.10–3.10 × 10^9^/L	2260	1.77	1.35–2.29
Lowest ALC during admission, x 10^9^/L Laboratory reference range for normal: 1.10–3.10 × 10^9^/L	2260	1.72	1.30–2.23
ANC (absolute neutrophil count) on admission, x10^9^/L Laboratory reference range for normal: 1.90–6.60 × 10^9^/L	2260	3.55	2.71–4.64
Highest ANC during admission, x 10^9^/L Laboratory reference range for normal: 1.90–6.60 × 10^9^/L	2260	3.66	2.82–4.76
C-reactive protein (CRP) on admission, mg/L Laboratory reference range for normal: 0.0- 5.0 mg/L	2231	3.40	1.30–7.70
Highest CRP during admission, mg/L Laboratory reference range for normal: 0.0–5.0 mg/L	2231	3.60	1.40–8.20
Lactate dehydrogenase (LDH) on admission, U/L Laboratory reference range for normal: 270–550 U/L	2231	381	340–436
Highest LDH during admission, U/L Laboratory range: 270–550 U/L	2231	383	342–440
**Chronic diseases**			
(1) **Diabetes mellitus (DM)**	**Median or N**		**IQR or%** **Denominator is tests done if available or the entire cohort**
HbA1c %(*n* = 123 tests done)	7.4		IQR:6.0–10.1
HbA1c ≥ 6.5 % (*n* = 123 tests done)	79		64.2
Diagnosed with DM inpatient based on any of the following: Charlson’s scoring for DM, discharge diagnosis, inpatient/outpatient DM medications and/or insulin (excluding short-acting insulin i.e. Actrapid), or laboratory criteria HbA1c ≥ 6.5 %	99		4.0
Known DM pre-admission based on DM meds/insulin prescribed within the first 2 days of admission, except for Actrapid	77		3.1
Discharged with DM medication AND insulin	7		0.3
Discharged with DM medication oral hypoglycaemic agents) alone	77		3.1
Discharged without any DM medications or insulin	15		0.6
(2) **Hyperlipidaemia**	**Median or N**		**IQR or%**
Total Cholesterol, mmol/L (*n* = 80 tests)	4.80		4.35- 5.45
HDL Cholesterol, mmol/L (*n* = 80 tests)	0.80		0.70–1.00
LDL Cholesterol, mmol/L (*n* = 77, not measurable in 3 due to high TG)	3.00		2.40–3.50
Triglycerides (TG), mmol/L (*n* = 80 tests)	1.90		1.50–2.60
Diagnosed with hyperlipidaemia inpatient based on any of the following: discharge diagnosis, inpatient, outpatient medications and medications on discharge for hyperlipidaemia. Measured lipid levels were not used to determine diagnosis of hyperlipidaemia since there are multiple individual-level risk factors influencing lipid-lowering targets which clinicians would assess.	69		2.8
Known hyperlipidaemia pre-admission based on medications for hyperlipidaemia prescribed within the first two days of admission and continued upon discharge.	43		1.7
(3) **Hypertension**	**N**		**%**
Diagnosed with hypertension inpatient based on discharge diagnosis and anti-hypertensive medications prescribed on discharge	135		5.4
Known hypertension pre-admission based on anti-hypertensive medications prescribed within the first two days of admission AND prescribed anti-hypertensive medication on discharge	114		4.6
(4) **Cardiovascular/cerebrovascular disease (IHD, AMI, CVA, Stroke)**	**N**		**%**
Diagnosed with cardiovascular / cerebrovascular disease inpatient based on discharge diagnosis, inpatient/outpatient medications	23		0.9
Known cardiovascular/cerebrovascular disease based on medications prescribed within the first two days of admission	3		0.1
Discharged with cardiovascular/cerebrovascular disease medications	17		0.7
(5) **Asthma/Chronic lung disease**	**N**		**%**
Diagnosed with asthma/chronic lung disease based on discharge diagnosis codes and inpatient/outpatient medications prescribed e.g. inhalers.	28		1.1
Known asthma/chronic lung disease based on medications prescribed within the first two days of admission	0		0.0
Discharged with inhalers	23		0.9
(6) **Mental health/psychiatric disorders**	**N**		**%**
Diagnosed with mental health/psychiatric disorder inpatient based on discharge diagnosis, inpatient/outpatient medications	24		1.0
Known mental health/psychiatric disorder inpatient based on medications prescribed within the first two days of admission AND prescribed on discharge	5		0.2
Discharged with psychiatric medications outpatient	9		0.4

**Table 2:** Clinical course synopsis for MWs (*n* = 16) who required a higher level of care in Intensive Care Unit (ICU)/High Dependency (HD) or oxygen supplementation in the ward.

Age range of participant ¶ (years)	Country of Origin	Duration and nature of symptoms at the time of admission (days)	Length of Hospital stay (LOS)	Known co-morbidities on admission	Newly identified co-morbidities	LOS in ICU (days)	Reason for ICU admission/higher care	Interventions in ICU or General Ward	Complications	Outcome including discharge destination
30–40	Bangladesh	1 day for AMS, day 20 of COVID-19	33	Pre-existing bipolar disorder on intermittent medication	Mania	1	Monitoring due to mental status	CT head and Lumbar puncture normal	None	Recovery facility
40–50	China	4; CS	20	None	None	5	Type 1 RF, ARDS	High flow oxygen (optiflow 40–50 L/min FiO2 40 %-50 %), awake proning, remdesivir	Bacterial pneumonia requiring AB	Recovery facility
50–60	China	8; CS	18	None	None	11	Type 1 RF	High flow oxygen (optiflow 40–60 L/min FiO2 40–60 %), awake proning, and remdesivir	Bacterial pneumonia requiring AB	Recovery facility
30–40	Bangladesh	4; CS	17	None	None	3	Type 1 RF	High flow oxygen (Venti Mask FiO2 35 %), awake proning, Etoricoxib	Bacterial pneumonia requiring AB	Recovery facility
50–60	Myanmar	4; CS	66	Dilated cardiomyopathy lost to follow-up, echocardiogram 3 years prior: LVEF 45 % with multiple RWMA	Echocardiogram during admission: LVEF 20 %, severe global hypokinesia. MIBI normal myocardial perfusion.	23	Type 1 RF, ARDS	High flow oxygen followed by intubation/ventilation, inotropes for septic shock, remdesivir	VAP, Septic shock, new-onset Atrial fibrillation, and an episode of SVT	Inpatient rehabilitation
30–40	Bangladesh	2 days of symptoms, both for index admission and re-admission; CS and chest pain (index) and chest pain (readmission)	9 days each for index admission and re-admission.	None	IHD, presented with NSTEMI	4 (index) and 3 (re-admission)	NSTEMI (index) and unstable angina (readmission)	Medical treatment, telemetry, dual antiplatelets (index). On re-admission, coronary angiogram- PCI to right coronary artery also performed.	None	Recovery facility and outpatient follow-up.
30–40	Bangladesh	1; CS, palpitations, and loss of consciousness	9	None	Paroxysmal Atrial fibrillation	2	Monitoring	Telemetry monitoring	None	Recovery facility, and outpatient cardiology follow-up
40–50	China	4; CS and dyspnea	22	None	None	3	Type 1 RF	NC oxygen supplementation in ward and ICU maximal FiO2 Venti Mask 35 %	Bacterial pneumonia requiring AB	Recovery facility
40–50	India	5; CS	12	None	None	NA	Type 1 RF	NC oxygen supplementation.	AB for bacterial pneumonia	Recovery facility
40–50	Bangladesh	3; CS	20	None	None	NA	Type 1 RF	NC oxygen supplementation and 10 days remdesivir (clinical trial), awake proning	None	Recovery facility
30–40	India	5; CS	13	None	None	NA	Type 1 RF	NC oxygen supplementation with awake proning, Etoricoxib.	None	Recovery facility
30–40	Bangladesh	6; CS and pleuritic chest pain	17	None	Newly diagnosed DM in the outpatient visit after discharge	NA	Type 1 RF	NC oxygen supplementation with awake proning, Etoricoxib.	None	Dormitory
20–30	Bangladesh	7 days (index) with CS and 2 days (readmission) with headache	20 days (index) and 3 days (readmission)	None	None (index), diagnosed with migraine with visual aura (readmission)	NA	Type 1 RF	NC oxygen supplementation with awake proning (index admission). CT brain unremarkable (second admission).	None	Recovery facility (index) and dormitory (readmission)
30–40	India	3 days (index) with CS, 5 days (first readmission) with new onset fever and chest pain, 2 days (second readmission) with fever and headache	5 days (index), 30 days (first readmission), 14 days (second readmission) 14	None (index), Hypertension, (first readmission), Hypertension and DM (second readmission)	Hypertension (index admission), DM- HbA1c 8.0 %, and hypertriglyceridemia (first readmission), no further new diagnoses (second readmission)	8 days (first readmission)	Myo-pericarditis with pericardial effusion, hypertensive crisis with acute pulmonary edema (first readmission)	First readmission: telemetry and intra-nasal cannula oxygen supplementation, and then needed intubation and peri‑cardiocentesis.	Polymicrobial bacteremia and *Staphyloccus. lugdenensis, Staphylococcus. epidermidis, Enterbacter cloacae* myo-pericarditis requiring AB	Recovery facility (index) and dormitory (readmissions)
40–50	Bangladesh	3; CS	19	None	DM- HbA1c 7.6 % Iron deficiency anemia, possibly dietary	NA	Type 1 RF	NC oxygen supplementation in the ward and 10 days remdesivir (clinical trial)	Bacterial pneumonia requiring AB	Recovery facility, outpatient follow-up for DM and anemia.
40–50	Malaysia (Indian ethnicity)	2; CS	23	DM- HbA1c 11.2 % Hypertension	None	NA	Type 1 RF	NC oxygen supplementation in the ward and 10 days remdesivir (clinical trial).	Bacterial pneumonia and *Enterobacter cloacae* bacteremia requiring AB.	Own home. Outpatient follow-up in Malaysia for DM and Hypertension.

***Footnotes:***.

***¶****There are only 16 MWs represented here in this table, hence age of participant is represented in the decade rather than actual age to avoid any inadvertent identification****.***

***AB****- refers to systemic antibiotics most commonly IV piperacillin-tazobactam, vancomycin, and amoxicillin-clavulanic acid for suspected/proven secondary bacterial infections such as pneumonia, bacteremia, etc.****AMS:****Altered mental state.****ARDS****: Acute Respiratory Distress Syndrome.****CS:****COVID-19-related symptoms may include one or more of the following: fever, cough, rhinorrhea, nasal congestion, sore throat, and myalgia. Other symptoms are specified.****CT****: Computed tomography scan.****DAPT:****Dual Antiplatelet therapy.****DM:****Diabetes mellitus****. FiO2:****fractional inhaled oxygen.****IHD****: Ischemic Heart Disease.****LOS****: length of stay.****LVEF****: Left Ventricular Ejection Fraction.****MIBI****: Myocardial perfusion scan (Methoxyisobutylisonitrile).****NC****: Nasal cannula.****NSTEMI****: non-ST elevation Myocardial infarction. LOS: Length of Stay.****O2****: refers to supplementary oxygen.****PCI:****Percutaneous coronary intervention.****RF****: Respiratory Failure.****RWMA:****Regional Wall Motion Abnormalities.****SVT:****Supra-ventricular tachycardia.****VAP****: Ventilator associated pneumonia.*

### 3.2 Prevalence of chronic diseases in MWs and comparison of COVID-19 outcomes

247 (9.8 %) MWs had at least one chronic disease. Table 1 shows the prevalence of chronic diseases, including the proportion that were newly diagnosed. Notably, 99 (4.0 %) had diabetes mellitus, newly diagnosed in 22 (22.2 %), 69 (2.8 %) had hyperlipidaemia, newly diagnosed in 26 (37.7 %), and 135 (5.4 %) had hypertension, newly diagnosed in 21 (15.5 %). The prevalence of cardiovascular and cerebrovascular disease was low (<1.0 %). 24 (1.0 %) were diagnosed with mental health/psychiatric disorders and 5 (0.2 %) were taking psychiatric medications at the time of admission.

We compared the 247 MWs who had at least one chronic disease compared to the 2251 MWs without chronic disease. The bivariate unadjusted analysis is shown in Table 3. Those with chronic diseases were older and more likely to be of Indian origin. A higher proportion of those with chronic disease had radiographic pneumonia compared to those without; 17.6 % (43/244) vs 10.7 % (235/2200), *p* < 0.01.

**Table 3:** Comparison of MWs with at least one chronic disease a with those without.

		**With chronic disease(s)** **(*n*=247)**		**Without chronic disease(s)** **(*n*=2251)**				
**Descriptive Characteristics**		**N or median**	**% or IQR**	**N or median**			**% or IQR**	**P value**
Age (years)		43	36–48	33			28–38	<0.01
Nationality	Bangladeshi	132	53.4	1527			67.8	<0.01
	Indian	84	34.0	550			24.4	
	Chinese	10	4.0	101			4.5	
	Thai	3	1.2	15			0.7	
	Others	18	7.3	58			2.6	
Housing	Dormitory	239	96.8	2200			97.7	0.339
	Non-dormitory	8	3.2	51			2.3	
Charlson’s co-morbidity Index score		0	IQR: 0–1 Range:0–4	0			IQR: 0–0 Range:0–3	<0.01
LOS for index admission for COVID-19 (days)		6	5–9	5			3–6	<0.01
Required ICU/HD care (Yes)		2	0.8 %	6			0.3	0.152
**COVID-19 related radiology**	**Tests done**	**N**	**%** **Denominator is tests done**	**Tests done**		**N**	**%** **Denominator is tests done**	**P value**
Abnormal CXR within the first 2 days of admission	244	43	17.6	2200		235	10.7	<0.01
Abnormal CT thorax during admission	6	3	50.0	11		4	36.4	0.692
Normal CXR in the first 2 days of admission with subsequent abnormal CXR	201	8	4.0	1965		21	1.1	<0.01
**COVID-19 related biomarkers ¶**	**Tests done**	**Median**	**IQR**	**Tests done**		**Median**	**IQR**	**P value**
ALC on admission x10^9^/L	239	1.77	1.34–2.26	2021		1.77	1.35–2.30	0.858
Lowest ALC during admission, x 10^9^/L	239	1.67	1.25–2.15	2021		1.73	1.30–2.25	0.194
ANC on admission, x 10^9^/L	239	3.61	2.61–4.89	2021		3.55	2.71–4.60	0.738
Highest ANC during admission, x 10^9^/L	239	3.97	2.87–5.10	2021		3.64	2.81–4.70	0.025
CRP on admission, mg/L	236	4.0	2.0–10.0	1995		3.3	1.3–7.5	<0.01
Highest CRP during admission, mg/L	236	4.9	2.1–12.6	1995		3.4	1.3–7.9	<0.01
LDH on admission, U/L	236	381	344–451	1995		381	340–435	0.424
Highest LDH during admission, U/L	236	390	348–461	1995		383	341–439	0.137
**Co-infections †**	**Tests done**	**N**	**%** **Denominator is sub-cohort, *n*=247**	**Tests done**		**N**	**%** **Denominator is sub-cohort, *n*=2251**	**P value**
Bacterial bloodstream infection (positive blood culture)	17	1	0.4	45		1	0.0	<0.01
TB (Mycobacterium TB PCR positive)	7	1	0.4	29		1	0.0	0.057
Acute dengue infection as defined by positive Dengue NS1 antigen	14	0	0.0	134		3	0.1	0.566
**COVID-19-related therapeutics including those given via clinical trial or off-label use**		**N**	**% Denominator, *n*=247**	**N**			**% Denominator, *n*=2251**	**P value**
Remdesivir		3	1.2	3			0.1	<0.01
Hydroxychloroquine		3	1.2	6			0.3	<0.01
Prescribed steroids inpatient (as defined by dexamethasone, prednisolone, hydrocortisone)		8	3.2	2			0.1	<0.01
Prescribed COX-2 inhibitor (Etoricoxib) inpatient		14	5.7	113			5.0	0.66
**Outcomes from index COVID-19 admission**		**N**	**%**		**N**		**%**	**P value**
Readmitted within 30 days		8	3.2	45			2.0	0.199

a MWs with at least one chronic disease from the following list: DM, hypertension, hyperlipidaemia, ischemic heart disease/cerebrovascular disease, asthma/chronic lung disease, mental health/psychiatric disorders ¶ ALC: Absolute lymphocyte count. ANC: Absolute neutrophil count. LDH: Lactate dehydrogenase. CRP: C- reactive protein. † These refer to co-infections with a microbiological diagnosis.

The multivariable logistic regression model (Table 4) showed Charlson’s score ≥ 1 (compared to 0) was associated with 80 % higher odds of moderate-severe COVID-19 outcome, aOR (adjusted odds ratio) 1.83 (95 % CI 1.16–2.90), *p* = 0.01, after adjusting for age and nationality.

**Table 4:** Multivariable logistic regression of factors associated with moderate to severe COVID-19 outcome, *n* = 2498.

**Variable**		**Adjusted odds ratio (95% CI)**	**P value**
Charlson’s score	Charlson’s score ≥ 1	1.83 (1.16–2.90)	0.01
	Charlson’s score=0	1.00 (reference)	
Age		1.04 (1.02–1.06)	<0.001
Nationality	Bangladeshi	0.57 (0.38–0.85)	0.01
	Indian	0.61 (0.39- 0.94)	0.02
	Others	1.00 (reference)	–

## 4 Discussion

### 4.1 Explaining and interpreting the results in the context of other evidence

In this large study of MWs with symptomatic COVID-19 in Singapore, we observed overall good clinical outcomes during their index admission; few patients required ICU/HD admission and there was no mortality. There could be many reasons for this, such as younger age, the healthy worker effect, and proactive public health policies facilitating early access to medical care during the outbreak. The national response led by the multi-ministry task force, with close collaboration by the Ministry of Health and Ministry of Manpower allowed early identification of COVID-19 with enhanced surveillance in dormitories with testing conducted on-site with early referral for hospital admission for PCR-positive MWs. (Tan et al., 2021) Multivariable regression model shows higher Charlson’s score was associated with higher odds of moderate-severe COVID-19 after adjusting for age and nationality.

To our knowledge, this is the largest study to examine the prevalence of chronic disease in this population in Singapore, where we detected a rate of between 2.8 % (for hyperlipidaemia) to 5.4 % (hypertension) prevalence of chronic metabolic diseases, in a relatively young, male, MW population predominantly from the Indian subcontinent. There had been a paucity of data of chronic disease prevalence in MWs in Singapore prior to the pandemic, representing a knowledge gap that led to this study. Our institution managed a large proportion of the MWs with symptomatic COVID-19 in Singapore during the outbreak. Of the chronic diseases we studied, a significant proportion were newly diagnosed (15.5 %, hypertension, 22.2 % diabetes mellitus, 37.7 % hyperlipidemia). While comparison has limitations, the prevalence of chronic diseases in MWs was either similar to or higher than the self-reported disease prevalence in Singaporeans aged 30–39 (National Population Health Survey, 2019), specifically for diabetes mellitus, 4.0 % vs 0.9 % (MWs vs Singaporeans of both genders), hyperlipidaemia, 2.8 % vs 3.2 % (MWs vs Singaporean men). (Ministry of Health S, 2020) In a study at Changi General Hospital in Singapore, Tee *et al*. found that 8.8 % of 240 male MWs with COVID-19 had pre-diabetes and 7.9 % had diabetes mellitus. (Tee et al., 2020) However, the MWs in that study were generally older with mean age of 44.2 years (SD 8.5) when compared to this cohort of relatively younger MWs with mean age of 34.9 years (SD 7.8), median age 34 years; ; ; and only 25 % being older than 40 years. (Tee et al., 2020) The relative difference in the age range of individuals in the two studies may explain the higher DM prevalence in the study by Tee *et al*. Similarly, a study from Singapore General Hospital in 883 MWs with mild or asymptomatic COVID-19 with a median age of 45 years found that 7.1 % had diabetes mellitus, 5.9 % had hyperlipidaemia and 15.9 % had hypertension. The prevalence of chronic metabolic diseases was higher in MWs of South Asian origin. However, the authors used different criteria to attribute a diagnosis of chronic disease e.g., those who had blood pressure persistently above 140/90 mmHg were classified as hypertensive regardless of the need for anti-hypertensive medications. (Mattar et al., 2022)

Our study indicates a significant burden of chronic disease amongst MWs despite our relatively young cohort, but we may have underestimated the true prevalence of chronic diseases. The healthy worker effect assumption may not necessarily hold and numerous reasons may explain the burden of chronic diseases in this population. (Razum, 2008; Aldridge et al., 2018) Firstly, the social determinants of health, specifically an individual’s socioeconomic status, living accommodations, occupation, access to food, and social inclusion (or lack thereof) can have important impacts on health and chronic disease. (Marmot, 2007; Marmot, 2005) Emerging evidence suggests that chronic psychosocial stress may contribute to obesity, and metabolic diseases through rising stress hormones, pro-inflammatory cytokines, and epigenetic changes. (Swinburn et al., 2011; Ford et al., 2017; Ouakinin et al., 2018) A cross-sectional study by Ang *et al*. showed a high level of psychological stress amongst MWs in Singapore even before the pandemic linked to debt, language/cultural barriers, and financial stress. (Ang et al., 2017) MWs and doctors alike had insufficient knowledge about MWs’ entitled access to health care before the pandemic. (Ang et al., 2017; Ang et al., 2020) At the time of our study, health care access did not include chronic disease screening and management, and availability was at the discretion and goodwill of the employer. MWs may have obtained medications from low-cost local NGO clinics or their country of origin with supply chains disrupted in the pandemic. Notably, as of 1st April 2022, statutory changes in Singapore have allowed MWs in the CMP sector primary care access with the introduction of compulsory insurance for MWs in a capitation model supported by the employer with a small individual co-pay by the MW at the time of utilisation. (Ministry of Manpower, 2022)

### 4.2 Strengths and limitations of the study

The strengths of this study are our large data set and ability to examine the clinical course and complications not just for the index COVID-19 admission but also for early re-admission where we expect to see early complications from COVID-19. The re-admission rate and prevalence of these complications were low in our study. Secondly, we used a combination of data points specifically diagnosis codes, medication prescriptions, and laboratory data where relevant to attribute a diagnosis of a specific chronic disease. While misclassification bias may still exist, we expect this approach would have minimized it. Thirdly, we defined our study period from 1st to 30th April 2020 to reduce ascertainment bias for COVID-19 as the PCR-positive MWs admitted then were symptomatic cases. There were higher numbers of asymptomatic cases of SARS-CoV-2 infection admitted from May 2020 onwards due to active case finding. One of our aims was to examine the association of chronic disease on COVID-19 disease severity, hence including asymptomatic PCR-positive MWs identified via active case finding would have resulted in a biased denominator and affected the validity of the association of chronic disease on COVID-19 disease severity. Fourthly, our facility was the main centre admitting adult COVID-19 patients during the time- period of the study enhancing the confidence of data completeness for early post-discharge complications with a low chance of missing data.

There are limitations to our study. Firstly, there could be case ascertainment bias and under-estimation of chronic disease as age-appropriate laboratory screening for chronic disease was based on clinician judgement. We used the criteria of medication prescribed within 2 days of admission as a surrogate for previously known diseases. However, we may have residual information bias, although likely to be non-differential. Secondly, there are limitations to the generalizability of our findings, particularly for chronic disease prevalence as the MWs infected in the first part of the outbreak (and thus included in our study) may not be representative of the entire MW population in Singapore although representative of those residing in high-density dormitories. Well-designed cohort studies in the migrant population are needed to systematically assess disease prevalence, incidence, and outcomes. Thirdly, our assignment of ‘previously known’ chronic disease does not necessarily mean the MWs were on medications prescribed by doctors in the Singapore healthcare system. They may have had chronic medications prescribed by doctors in their country of origin and our data sources are unable to determine this detail though the routine medication-reconciliation process during the hospital admission is meant to determine this. Fourthly, while we describe 1.0 % MWs diagnosed with mental health disorders during the index admission, we think this is an underestimate. We expect only a fraction experiencing psychological stress would have been referred to a psychiatrist or prescribed psychiatric medications due to factors such as cultural stigma, language barriers despite the availability of translators, and lack of mental health literacy in both MWs and possibly clinicians. Psychological stress may also manifest only weeks or months later. (Chan and Kuan, 2020) Other quantitative and qualitative studies have described the stress, anxiety, and depression faced by MWs from not being able to work, financial worries, prolonged isolation, and uncertainty. (Saw et al., 2021; Yee et al., 2021)

### 4.3 Policy implications and conclusions

In conclusion, despite good COVID-19 morbidity and mortality outcomes in this MW COVID-19 outbreak, our study and others underscore the importance of developing public healthcare policies to ensure MWs have reliable and affordable access to outpatient care, preventive/primary care, and chronic disease management. Our study revealed a high proportion of chronic diseases that were newly identified during the index COVID-19 admission. The access to outpatient primary care and chronic disease care needs to come with enhanced integration with the healthcare system and measures to address the wider social determinants of health. The WHO Health and Migration Program calls for universal health coverage and migrant-inclusive policies as an important goal in the care of non-communicable diseases. (Geneva: World Health Organisation, 2022) There are >222 million migrants globally, and in Singapore, they comprise 37 % of our working population. (Rajaraman et al., 2020) Under Singapore law, employers are ultimately responsible for the healthcare of low-wage MWs. The COVID-19 pandemic brought to the fore issues of access disparities due to gaps in outpatient care, health delivery models, and healthcare financing, and catalyzed further efforts in bridging these gaps by policymakers, employers, and civil society. Importantly, the changes implemented for mandatory outpatient coverage for the large segment of low-wage MWs in the CMP sector are anticipated to improve health-seeking behavior and access. Amplifying MW awareness of their occupational and health benefits/entitlements, improving employer engagement, and healthcare provider/institutions’ language and cultural competencies are important to address the barriers that may limit access to care even if it was available. While dense housing remains a key vulnerability, the Ministries of Manpower, Health, and National Development have reviewed and embarked on new benchmarks for housing accommodations for MWs, but it will likely take several years to replace the current dormitories. (Ministry of National Development Press Release, 2022)

Furthermore, we postulate the collective benefit of granting MWs systematic access to outpatient chronic disease, preventive care may outweigh the consequences of not doing so, such as the economic and social impact Singapore faced during the ‘circuit breaker’ period as an example. In a white paper published by the Singapore government in 2023, the COVID-19 outbreak impacting MWs in the dormitories was cited as an important lesson, specifically a key reflection was the need to offer more comprehensive medical care to MWs. (COVID-19 White Paper, 2024) A primary care health plan for MWs in has since been implemented. (Ministry of Manpower, 2024) This is a unique opportunity to review public health policies for this population as disease outbreaks and events that affect our MWs impact occupational justice and dignity while also impacting society at large. (Sadarangani SP et al., 2017; Ang et al., 2017) The time is right for a more inclusive narrative for migrant health in Singapore and our experience may prove valuable for other countries receiving economic migrants.

## Funding

No specific funding was needed in the conduct of this study.

## CRediT authorship contribution statement

**Sapna P. Sadarangani:** Conceptualization, Methodology, Writing – original draft, Data curation, Supervision, Project administration, Writing – review & editing. **Joshua K. Tan:** Data curation, Project administration, Writing – review & editing, Methodology, Writing – original draft, Formal analysis, Software. **Pei Hua Lee:** Project administration, Formal analysis, Writing – review & editing, Data curation, Writing – original draft. **Hanley Ho:** Conceptualization, Supervision, Software, Data curation, Writing – review & editing. **Angela Chow:** Formal analysis, Writing – review & editing, Conceptualization, Supervision, Software. **Rinkoo Dalan:** Writing – review & editing, Methodology, Conceptualization. **Lai Gwen Chan:** Writing – review & editing, Methodology. **Thirugnanam Umapathi:** Methodology, Writing – review & editing, Supervision. **Shawn Vasoo:** Conceptualization, Writing – review & editing, Supervision, Resources.

## Declaration of competing interest

The authors declare that they have no known competing financial interests or personal relationships that could have appeared to influence the work reported in this paper.
